# Using Gross Energy Improves Metabolizable Energy Predictive Equations for Pet Foods Whereas Undigested Protein and Fiber Content Predict Stool Quality

**DOI:** 10.1371/journal.pone.0054405

**Published:** 2013-01-14

**Authors:** Jean A. Hall, Lynda D. Melendez, Dennis E. Jewell

**Affiliations:** 1 Department of Biomedical Sciences, College of Veterinary Medicine, Oregon State University, Corvallis, Oregon, United States of America; 2 Pet Nutrition Center, Hill's Pet Nutrition, Inc, Topeka, Kansas, United States of America; Auburn University, United States of America

## Abstract

Because animal studies are labor intensive, predictive equations are used extensively for calculating metabolizable energy (ME) concentrations of dog and cat pet foods. The objective of this retrospective review of digestibility studies, which were conducted over a 7-year period and based upon Association of American Feed Control Officials (AAFCO) feeding protocols, was to compare the accuracy and precision of equations developed from these animal feeding studies to commonly used predictive equations. Feeding studies in dogs and cats (331 and 227 studies, respectively) showed that equations using modified Atwater factors accurately predict ME concentrations in dog and cat pet foods (r^2^ = 0.97 and 0.98, respectively). The National Research Council (NRC) equations also accurately predicted ME concentrations in pet foods (r^2^ = 0.97 for dog and cat foods). For dogs, these equations resulted in an average estimate of ME within 0.16% and 2.24% of the actual ME measured (equations using modified Atwater factors and NRC equations, respectively); for cats these equations resulted in an average estimate of ME within 1.57% and 1.80% of the actual ME measured. However, better predictions of dietary ME in dog and cat pet foods were achieved using equations based on analysis of gross energy (GE) and new factors for moisture, protein, fat and fiber. When this was done there was less than 0.01% difference between the measured ME and the average predicted ME (r^2^ = 0.99 and 1.00 in dogs and cats, respectively) whereas the absolute value of the difference between measured and predicted was reduced by approximately 50% in dogs and 60% in cats. Stool quality, which was measured by stool score, was influenced positively when dietary protein digestibility was high and fiber digestibility was low. In conclusion, using GE improves predictive equations for ME content of dog and cat pet foods. Nondigestible protein and fiber content of diets predicts stool quality.

## Introduction

Most pet owners in the United States feed their pets commercially prepared pet foods. Fulfilling the nutrient requirements of pet animals with commercially prepared foods requires knowledge about the food as well as an understanding of the lifestage nutritional needs of the pet. For example, determination of a pet's energy requirements for a particular age and physiologic state can be calculated [1]. To determine how much food to feed, one must know the energy density of the food. Dividing the energy requirement of the pet by the energy density of the food determines the daily amount to feed. Thus, knowing the energy density of a food is important in determining the quantity of food that is offered each day. Because pets eat to maintain energy intake, energy density also determines the amount of all other nutrients that a pet ingests. Therefore, the non-energy nutrients in the food must be balanced relative to energy density to ensure adequate nutrient intake.

The gross energy (GE) in a food is defined as the total chemical energy measured from complete combustion of the food in a bomb calorimeter [2]. Digestible energy (DE) and metabolizable energy (ME) are the more typical terms used in canine and feline nutrition. Digestible energy refers to GE minus energy lost in feces. Metabolizable energy refers to DE minus energy lost in urine plus energy lost as gaseous products of digestion. However, because methane production is negligible in dogs and cats [3], ME is usually defined as DE minus energy lost in urine.

The most accurate determination of the DE or ME content of food is obtained through animal feeding studies. The Association of American Feed Control Officials (AAFCO) [4] has published accepted protocols for the determination of ME of dog and cat foods. To determine DE, it is sufficient to know the GE consumed and to collect feces and calculate fecal energy losses. To determine ME one must collect urine as well as feces or calculate urine energy losses through knowledge of urinary nitrogen loss [4]. Because animal studies are labor intensive, predictive equations are used extensively for calculation of ME values [5]–[7]. The AFFCO [4] recommends a predictive equation based primarily on fixed energy values and digestibility coefficients for dietary components (crude protein, crude fat, and carbohydrate) for estimating the ME content of dog and cat foods. The original factors in the equation described by Atwater [8] were modified by Kendall et al. [9] for dogs and Kendall et al. [5] for cats. The modified Atwater factors for dogs and cats (3.5 kcal/g protein, 8.5 kcal/g fat, and 3.5 kcal/g carbohydrate) provide reasonable estimates of ME for commercial pet foods with digestibilities in the range of 75 to 85% [3]. Because the same formula is used for both dogs and cat foods, irrespective of the relative fiber content or presumed digestibility, calculations may underestimate energy content of highly digestible foods and overestimate those of less digestible foods [2], [10]. Underestimation of the ME content could result in overfeeding and contribute to obesity and its associated disorders [11]. Because of the possible inconsistency of the Atwater equation to predict ME, many researchers have tried to identify more accurate equations to estimate energy content of pet foods [2], [3], [11]. For example, predictive equations have been designed to take into account the fiber portion of the food [12]–[14], or the amino acid and non-amino acid compounds in the crude protein fraction to better predict ME [11], [15].

The first objective of this retrospective review of 558 digestibility studies in dogs and cats, which were conducted over a 7-year period and based upon AAFCO feeding protocols, was to compare the accuracy and precision of equations developed from these digestibility studies to equations that use modified Atwater factors to predict ME concentration of dog and cat pet foods. In addition, equations developed from these digestibility studies were also compared to NRC [2] predictive equations.

The second objective of this retrospective study was to examine the effects of nutrient digestibility on stool quality. Previous studies have suggested that an overabundance of protein in the diet may be a negative factor for stool quality, whereas dietary fiber is a positive factor [16]–[19]. These findings assume that excess protein or dietary fiber is passed into the large intestine providing substrate for microbial fermentation and growth. We hypothesized that stool quality is best when dietary protein digestibility is high and dietary fiber digestibility is low.

## Materials and Methods

### Dogs and Cats Ethics Statement

The study protocols were reviewed and approved by the Institutional Animal Care and Use Committee, Hill's Pet Nutrition, Inc., Topeka, KS, USA (Permit Numbers: CP13, CP14). All dogs used in these studies were immunized against canine distemper, adenovirus, parvovirus, bordetella, and rabies. All cats were immunized against rabies, viral rhinotracheitis, feline calicivirus, and feline panleukopena virus. None had chronic systemic disease on the basis of results of an annual physical examination, complete blood count determination, serum biochemical analyses, and urinalysis. Dogs were housed individually in indoor runs and allowed exercise in groups. Cats were housed individually and allowed exercise in indoor runs. Dogs and cats had access to natural light that varied with seasonal changes. All dogs and cats were provided with regular opportunities for socialization and environmental enrichment. Dogs and cats experienced behavioral enrichment through daily interaction and play time with caretakers, and by daily opportunities to run and exercise, with access to toys. All animals were owned by the commercial funders of this research and/or their affiliates, who gave permission for their animals to be used in these digestibility studies.

Over a period of 7 years, 558 digestibility studies were conducted using healthy adult Beagle dogs (n = 331 studies) or healthy adult domestic short hair cats (n = 227 studies). Altogether, 124 dogs with mean age of 6.7 years (range 2 to 12 years) and 138 cats with mean age of 8.0 years (range 1 to 15 years) participated in these studies.

### Foods

Many different types of foods were studied, including dry and canned dog foods, and dry and canned cat foods, with varying nutrient compositions. Both commercial and non-commercial foods were tested. All foods met the requirements established by AAFCO for complete and balanced pet foods for adult dogs or cats.

### Study design and measurements

All digestibility studies followed the AAFCO [4] quantitative collection protocol. Each test used six adult dogs or cats. Feeding tests consisted of two phases. The first phase was a pre-collection period of at least seven days to allow acclimation of dogs or cats to the test food, and to adjust food intake, as needed, to maintain body weight. The second phase lasted 5 days (120 hours) and was used for total collection of feces. The amount of food offered during the second phase was held constant, and based upon the amount of food determined to maintain body weight in phase one. Water was available at all times.

Food analytical measurements for energy, moisture, protein, fat, fiber and ash were performed as outlined by AAFCO [4]. Food composition of the experimental foods was determined by a commercial laboratory (Eurofins Scientific, Inc., Des Moines, IA) using Association of Analytical Communities (AOAC) methods. Digestibility coefficients for dry matter, fat, nitrogen-free extract (NFE; carbohydrate) and fiber were all calculated as apparent digestibility [(consumed – fecal)/consumed]. In order to correct for endogenous protein appearance in the feces, calculated endogenous loss based on metabolic body size was subtracted from fecal protein resulting in a calculation of true protein digestibility [(consumed – {fecal – endogenous protein})/consumed] using the estimate of endogenous protein of Kendall et al. [9]. The ME calculation used the methods outlined in AAFCO whereby DE is measured and ME is then calculated using the correction factor for energy lost in urine for dogs (1.25× g protein absorbed) or cats (0.86× g protein absorbed).

Stool quality was evaluated based on a grading system of 1 to 5. The graders were trained and previously evaluated for both accuracy and precision. The graders were masked to the foods being fed. A grade of 1 was assigned to feces that did not have solid form and was more than 75% liquid. A grade of 2 was assigned to feces that were soft and mounded, and approximately 50% solid and 50% liquid. A grade of 3 was assigned to feces if it had some cylindrical shape and was more than 75% formed and solid. A grade of 4 was assigned to feces that were greater than 75% cylindrical and if more than 50% of the feces were firm. A grade of 5 was assigned to feces if it was cylindrically shaped and if more than 80% of the feces were firm. Stool was scored during the phase two collection period and all scores were averaged to obtain a single score per animal. All animal scores were than averaged to obtain a single score for each digestibility study.

The absolute amount of protein (or fiber) that was not absorbed from the food was calculated by multiplying the amount of protein (or fiber) in the food by the percentage that was not digested (100– percentage digested  =  percentage not digested). This calculation estimated the amount of protein (or fiber) that was available to enter into the large intestine. Foods were classified as high or low for this variable, with all those above the median being “high” and all those below the median being “low.”

### Statistical analyses

Analyses were performed using the general linear model in Statistical Analysis Software (SAS) version 9 (SAS Institute, Cary, NC) for all response variables. Multivariate regression analysis was used to determine the relationship between the factors for dietary moisture, protein, fat, crude fiber and energy that resulted in the least variation between estimated and actual measurements based on these data. The estimates of ME from the predictive equations were compared to actual measured values to evaluate accuracy (average of the residuals when predicted was subtracted from actual) and precision (the variation of the residuals).

The relationship between stool quality and nutrient digestibility was evaluated by comparing measurements of stool quality with digestibility coefficients for protein, fat, fiber and dry matter. Because the digestibility coefficients for protein and fiber were most significant (*P*<0.01) in influencing stool quality, they were further evaluated by multivariant analysis. The multivariant analysis was accomplished using species (dog or cat) and forms (dry or canned) as a class variable and measured dietary ME as a covariate. Mean separation of foods that were high in nondigestible protein or fiber content were compared using the SAS general linear model with species (dog or cat) and the protein or fiber classification (high or low, with regards to the amount in each food that was available to enter the large intestine) as discreet variables. Means separation was accomplished by Duncan's least significant difference test. Significance was concluded to exist at *P*≤0.05, and also reported if *P*≤0.01.

## Results

Food compositions of canine and feline foods tested in the animal feeding studies areshown in **Tables** **1** **and** **2**, respectively. As expected, cat foods tended to be higher in protein and fat compared with dog foods. The measured digestibility coefficients for canine and feline foods tested in the animal feeding studies are shown in **Tables** **3** **and** **4**, respectively. There was a difference (*P*≤0.01) in digestibility of protein, fat, and fiber for canine foods between the dry and canned foods with dry foods having greater protein and fat digestibility and lesser fiber digestibility. In feline foods, there was a difference (*P*≤0.01) in digestibility of dry matter, fat, carbohydrate, and energy with dry foods having greater dry matter, fat, carbohydrate, and energy digestibility compared with canned foods.

**Table 1:** Food composition, expressed as means and standard deviation (SD), of canine foods used in digestibility studies.*,†

	All Dog Foods		Dry Dog Foods		Canned Dog Foods	
	(n = 331 studies)		(n = 259 studies)		(n = 72 studies)	
	Mean	SD	Mean	SD	Mean	SD
**Moisture**	22.8	27.6	8.3	0.9	74.9	2.5
**Protein**	18.8	7.7	22.3	4.3	6.2	1.2
**Fat**	11.9	5.5	14.1	3.8	3.8	1.9
**Ash**	4.3	2.0	5.1	1.4	1.4	0.3
**Crude Fiber**	4.1	4.3	5.0	4.5	1.0	0.7
**Gross Energy, kcal/kg**	3860	1416	4595	263	1219	168

* All analytical values are expressed as percentage of food as fed, unless otherwise indicated.

† Food composition of the experimental foods was determined by a commercial laboratory (Eurofins Scientific, Inc., Des Moines, IA) using AOAC methods.

**Table 2:** Food composition, expressed as means and standard deviation (SD), of feline foods used in digestibility studies.*,†

	All Cat Foods		Dry Cat Foods		Canned Cat Foods	
	(n = 227 studies)		(n = 173 studies)		(n = 54 studies)	
	Mean	SD	Mean	SD	Mean	SD
**Moisture**	23.5	29.6	7.1	1.3	76.3	2.2
**Protein**	27.2	10.4	32.8	3.4	9.5	1.2
**Fat**	15.3	6.7	18.4	4.2	5.3	1.6
**Ash**	4.7	3.2	5.7	3.1	1.7	1.0
**Crude Fiber**	3.0	2.7	3.6	2.8	1.2	1.0
**Gross Energy, kcal/kg**	4139	1617	5028	299	1292	175

* All analytical values are expressed as percentage of food as fed, unless otherwise indicated.

† Food composition of the experimental foods was determined by a commercial laboratory (Eurofins Scientific, Inc., Des Moines, IA) using AOAC methods.

**Table 3:** Digestibility coefficients, expressed as means and standard deviation (SD), of canine foods used in digestibility studies.*

	All Dog Foods		Dry Dog Foods		Canned Dog Foods	
	(n = 331 studies)		(n = 259 studies)		(n = 72 studies)	
	Mean	SD	Mean	SD	Mean	SD
**Dry Matter**	83.1	6.7	82.9	7.1	83.9	4.6
**Protein**	89.4^a,b^	4.2	89.7^a^	4.2	88.0^b^	4.1
**Fat**	93.0^a,b^	3.5	93.5^a^	2.9	91.2^b^	4.8
**Carbohydrate**	90.5	5.9	90.8	5.6	89.5	6.7
**Fiber**	39.7^a,b^	19.5	38.3^a^	18.1	44.8^b^	23.5
**Energy**	85.8	6.2	86.0	6.5	85.2	4.7

* All analytical values are expressed as percentages.

a,b Means with different superscripts in the same row are different (*P*≤0.01).

**Table 4:** Digestibility coefficients, expressed as means and standard deviation (SD), of feline foods used in digestibility studies.*

	All Cat Foods		Dry Cat Foods		Canned Cat Foods	
	(n = 227 studies)		(n = 173 studies)		(n = 54 studies)	
	Mean	SD	Mean	SD	Mean	SD
**Dry Matter**	82.5^a,b^	4.7	83.1^a^	4.5	80.6^b^	6.0
**Protein**	93.1	3.1	93.0	2.9	93.5	3.8
**Fat**	91.0^a,b^	4.5	91.9^a^	3.1	88.2^b^	6.5
**Carbohydrate**	85.3^a,b^	13.0	89.1^a^	5.5	73.3^b^	20.7
**Fiber**	44.2	23.7	43.4	22.1	47.5	29.2
**Energy**	85.5^a,b^	5.1	86.3^a^	4.3	82.8^b^	6.5

* All analytical values are expressed as percentages.

a,b Means with different superscripts in the same row are different (*P*≤0.01).

The measured ME concentrations of dog and cat pet foodsand the estimated ME concentrations calculated by commonly used predictive equationsare shown in **Tables** **5** **and** **6**, respectively. Using the predictive equation with modified Atwater factors for dogs and cats (3.5 kcal/g protein, 8.5 kcal/g fat, and 3.5 kcal/g carbohydrate) as described by AAFCO [4] to calculate ME involves calculating a carbohydrate estimate for NFE, which is obtained by subtracting percent protein, fat, crude fiber, moisture, and ash from 100%. The ME is then estimated by summing 3.5× protein concentration, 3.5× carbohydrate concentration, and 8.5× fat concentration. The other commonly used predictive equation from NRC [2] estimates ME for dogs and cats by first calculating GE using the equation GE  = 5.7× g protein +9.4× g fat +4.1× (g NFE + g fiber). Energy digestibility coefficients are calculated for dogs as (91.2–1.43× percentage crude fiber in DM) and for cats as (87.9–0.88× percentage crude fiber in DM). These digestibility coefficients then allow calculation of DE in dogs and cats as DE  =  GE × percentage energy digestibility/100 and, subsequent calculation of ME in dogs as ME  =  DE – (1.04× g protein) and in cats as ME  =  DE – (0.77× g protein).

**Table 5:** Metabolizable energy (ME; kcal/kg; means and standard deviation, SD), were determined in canine digestibility studies and compared to those calculated using predictive equations.

	All Dog Foods		Dry Dog Foods		Canned Dog Foods	
	(n = 331 studies)		(n = 259 studies)		(n = 72 studies)	
	Mean	SD	Mean	SD	Mean	SD
**Measured ME**	3126	1120	3723	432	978	176
**Calculated ME using modified Atwater factors** a	3121	1140	3700	319	1034	164
**Delta** b	−5	206	−22	228	56	63
**Absolute Delta** c	148	143	170	153	69	48
**Calculated ME using NRC predictive equations** d	3056	1140	3620	427	1025	171
**Delta** b	−70	197	−103	208	47	74
**Absolute Delta** c	149	147	172	155	67	56
**Calculated ME using new predictive equation** e	3126	1195	3723	416	978	173
**Delta** b	0	104	0	117	0	34
**Absolute Delta** c	76	71	90	74	26	22

a Predicted ME using equation with modified Atwater factors [4].

b The difference between measured and estimated ME.

c The absolute value of the difference between measured and estimated ME.

d Predicted ME using NRC [2] equations.

e Predicted ME using equation developed from the experimental animal feeding studies.

**Table 6:** Metabolizable energy (ME; kcal/kg; means and standard deviation, SD), were determined in feline digestibility studies and compared to those calculated using predictive equations.

	All Cat Foods		Dry Cat Foods		Canned Cat Foods	
	(n = 227 studies)		(n = 173 studies)		(n = 54 studies)	
	Mean	SD	Mean	SD	Mean	SD
**Measured ME**	3369	1374	4107	410	1005	189
**Calculated ME using modified Atwater factors** a	3316	1288	4017	306	1069	153
**Delta** b	−53	224	−90	241	64	81
**Absolute Delta** c	173	151	201	160	83	61
**Calculated ME using NRC predictive equations** d	3308	1297	4010	343	1059	153
**Delta** b	−61	240	−96	261	54	88
**Absolute Delta** c	180	170	210	182	86	55
**Calculated ME using new predictive equation** e	3369	1372	4107	395	1004	195
**Delta** b	0	82	0	90	−1	42
**Absolute Delta** c	63	52	71	55	35	29

a Predicted ME using equation with modified Atwater factors [4].

b The difference between measured and estimated ME.

c The absolute value of the difference between measured and estimated ME.

d Predicted ME using NRC [2] equations.

e Predicted ME using equation developed from the experimental animal feeding studies.

New equations for estimating ME in dogs and cats were derived from measured ME concentrations determined in the animal feeding studies. For the dog: ME  = 575+0.816× GE (kcal/kg) +12.08× percentage fat –52.76× percentage crude fiber –20.61× percentage protein –6.07× percentage moisture. For the cat: ME  = −541+0.923× GE (kcal/kg) +14.68× percentage fat –44.31× percentage crude fiber –4.21× percentage protein +4.80× percentage moisture.

**Figure 1:**
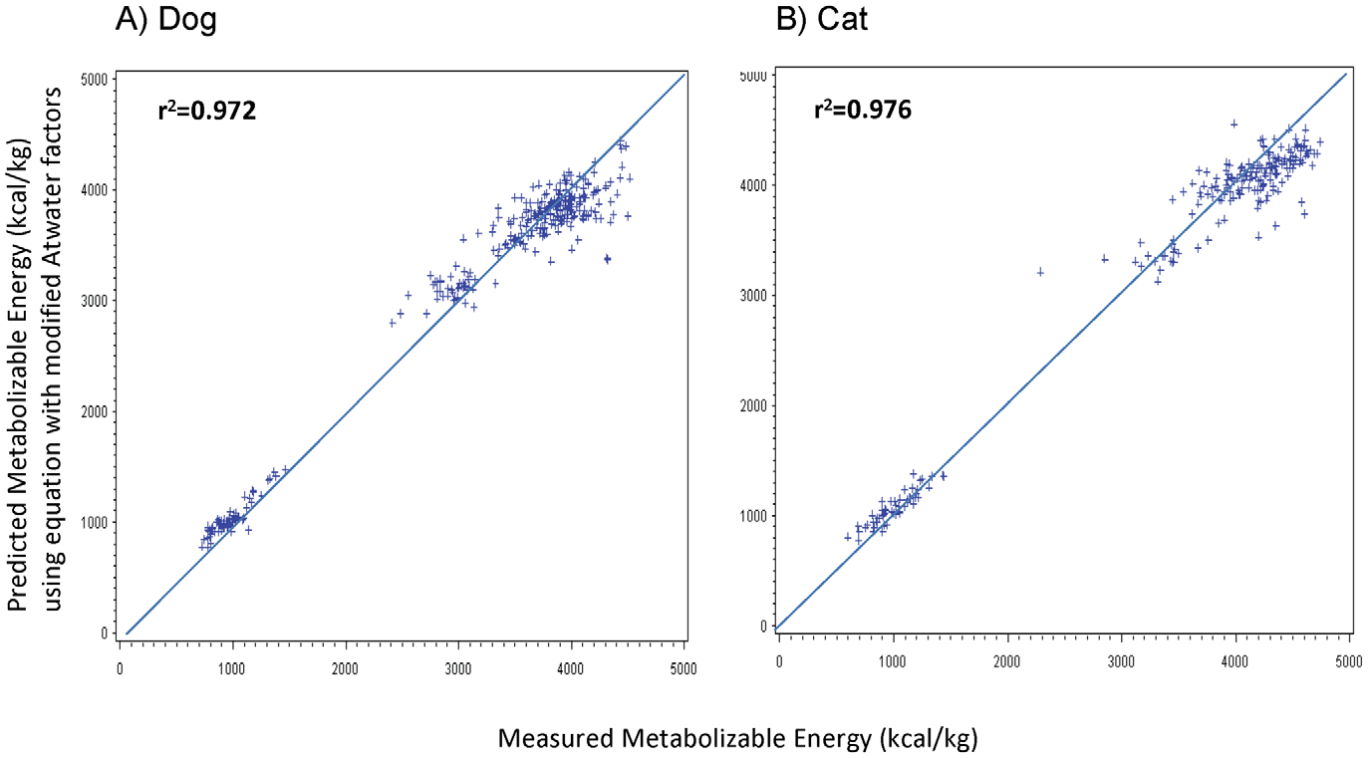
Relationship between measured metabolizable energy (ME) concentrations (x-axis) and ME concentrations predicted using equations with modified Atwater factors [4] (y-axis) for dog and cat pet foods. The modified Atwater factors are 3.5 kcal/g for protein and carbohydrate, and 8.5 kcal/g for fat. Ideally, all points should be on the line *x* = *y*. **A**) Measured ME concentrations were determined from 331 total digestibility studies in dogs, of which 259 used dry dog foods and 72 used canned dog foods. **B**) Measured ME concentrations were determined from 227 total digestibility studies in cats, of which 173 used dry cat foods and 54 used canned cat foods.

All ME concentrations that were calculated from predictive equations were on average less than 3% different from ME concentrations measured in animal feeding studies. In dogs, the average predicted ME difference from measured ME concentrations was significantly less than 1% for ME concentrations that were calculated using the equation with modified Atwater factors [4] or the equation generated from the study itself (**Table** **5**). In cats, the average predicted ME difference from measured ME concentrations was 2% for ME concentrations that were calculated using the equation with modified Atwater factors or the NRC [2] equations (**Table** **6**). Both the NRC equations and the equation using modified Atwater factors slightly underestimated ME concentrations in dry foods and overestimated ME concentrations in canned foods. The equations generated for dogs and cats from the animal feeding studies did not have this bias. The scatter plots comparing ME concentrations that were calculated from predictive equations vs. the actual ME concentrations that were measured in animal feeding studies are shown in **Figures** **1** **to** **3**. The r^2^ of ME concentrations determined by both the modified Atwater and NRC predictive equations exceeded 0.97, whereas ME concentrations determined by equations generated from the study itself, which utilized GE, had an r^2^ greater than 0.99.

**Figure 2:**
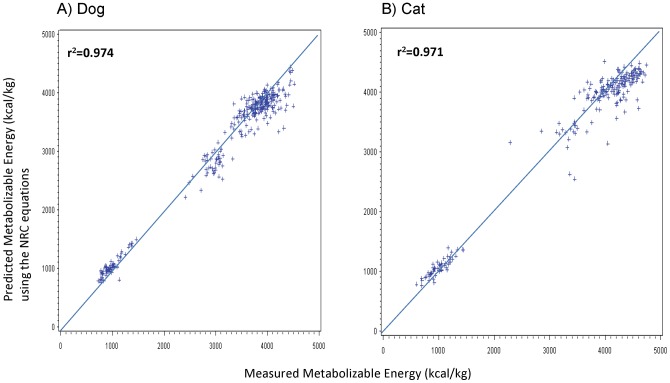
Relationship between measured metabolizable energy (ME) concentrations (x-axis) and ME concentrations predicted using National Research Council (NRC) equations [2] (y-axis) for dog and pet foods. Ideally, all points should be on the line *x* = *y*. **A**) Measured ME concentrations were determined from 331 total digestibility studies in dogs, of which 259 used dry dog foods and 72 used canned dog foods. The NRC equations for dogs first calculate gross energy (GE) using the equation GE  = 5.7× g protein +9.4× g fat +4.1× (g NFE + g fiber). Energy digestibility coefficients are then calculated for dogs as (91.2–1.43× percentage crude fiber in DM). These digestibility coefficients then allow calculation of digestible energy (DE) in dogs as DE  =  GE × percentage energy digestibility/100 and, subsequent calculation of ME as ME  =  DE – (1.04× g protein). **B**) Measured ME concentrations were determined from 227 total digestibility studies in cats, of which 173 used dry cat foods and 54 used canned cat foods. The NRC equations for cats first calculate GE using the equation GE  = 5.7× g protein +9.4× g fat +4.1× (g NFE + g fiber). Energy digestibility coefficients are then calculated for cats as (87.9–0.88× percentage crude fiber in DM). These digestibility coefficients then allow calculation of DE in cats as DE  =  GE × percentage energy digestibility/100 and, subsequent calculation of ME as ME  =  DE – (0.77× g protein).

**Figure 3:**
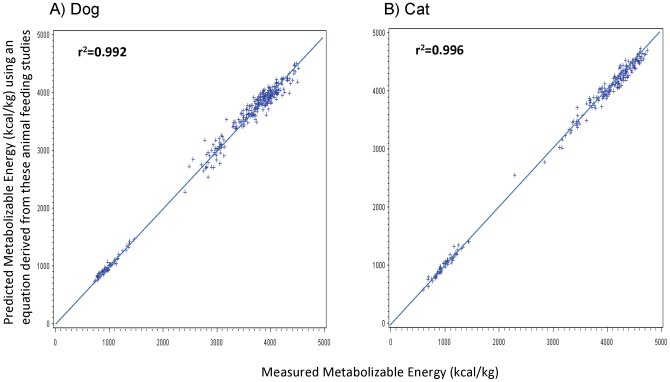
Relationship between measured metabolizable energy (ME) concentrations (x-axis) and ME concentrations predicted using new study-derived equations (y-axis) for dog and cat pet foods. New study-derived equations were derived from measured ME concentrations in the animal feeding studies. These equation sum coefficients multiplied by gross energy (GE), fat, crude fiber, protein, and moisture percentages. Ideally, all points should be on the line *x* = *y*. **A**) Measured ME concentrations were determined from 331 total digestibility studies in dogs, of which 259 used dry dog foods and 72 used canned dog foods. For the dog: ME  = 575+0.816× GE (kcal/kg) +12.08× percentage fat –52.76× percentage crude fiber –20.61× percentage protein –6.07× percentage moisture. **B**) Measured ME concentrations were determined from 227 total digestibility studies in cats, of which 173 used dry cat foods and 54 used canned cat foods. For the cat: ME  = −541+0.923× GE (kcal/kg) +14.68× percentage fat –44.31× percentage crude fiber –4.21× percentage protein +4.80× percentage moisture.

The influence of nutrient digestibility on stool quality, assessed using a 1 to 5 grading system, is shown in **Table 7**. In both dogs and cats, there was a positive effect on stool quality when they were fed foods that had lesser amounts of protein or greater amounts of fiber passing into the large intestine. A food with both a reduction in digestibility and an increase in concentration of dietary fiber had a significant positive effect (*P*<0.01) on stool quality. With regards to protein, there was a significant positive effect (*P*<0.01) on stool quality if protein digestibility of foods was increased and if a reduced amount of protein entered the large intestine. There was no correlation between dietary protein concentration in foods and stool quality.

**Table 7:** Canine and feline stool scores, expressed as means ± SEM, using a 1 to 5 grading system^*^ are shown for foods classified as either low or high† with regard to the amount of protein or fiber that was available to enter the large intestine.

Species	Large Intestinal Protein		Large Intestinal Fiber	
	Low†	High†	Low†	High†
**Canine** (n = 331 foods)	4.3±0.04^a^	4.1±0.04^b^	4.1±0.04^a^	4.2±0.04^b^
**Feline** (n = 227 foods)	4.1±0.05^a^	3.7±0.05^b^	3.8±0.06^a^	4.0±0.07^b^

* A grade of 1 was assigned to feces that did not have solid form and was more than 75% liquid. A grade of 2 was assigned to feces that was soft and mounded, and approximately 50% solid and 50% liquid. A grade of 3 was assigned to feces if it had some cylindrical shape and was more than 75% formed and solid. A grade of 4 was assigned to feces that were greater than 75% cylindrical and if more than 50% of the feces was firm. A grade of 5 was assigned to feces if it was cylindrically shaped and if more than 80% of the feces were firm.

† A high or low classification was assigned to each food based on the amount of dietary protein or fiber that was available to enter the large intestine. The absolute amount of protein (or fiber) that was not absorbed from each food was calculated by multiplying the amount of protein (or fiber) in each food by the percentage that was not digested (100– percentage digested  =  percentage not digested). This calculation estimated the amount of protein (or fiber) that was available to enter the large intestine for that food. Foods above the median value were classified as high for this variable, whereas foods below the median value were classified as low for this variable.

a,b Means with different superscripts within a row under the large intestinal protein or fiber columns are different (*P*≤0.05). The fiber effect was a main effect with no interaction with species, whereas the protein effect had a species by protein interaction and mean separation was completed independently within each species.

## Discussion

Animal feeding studies (331 in the dog and 227 in the cat), performed over a 7-year period that were based upon AAFCO feeding protocols, were reviewed to compare the accuracy and precision of equations developed from these digestibility studies to published predictive equations for ME concentrations in dog and cat pet foods. The use of the modified Atwater factors [4] predictive equation resulted in an accurate prediction of ME especially for dog foods, where there was only a 5 kcal/kg difference in the average predicted ME concentration compared with the measured ME concentration. In the cat, both the modified Atwater factors and the NRC [2] predictive equations were reasonably accurate. However, the ME prediction obtained using a new equation that takes into account measured GE plus specific coefficients for moisture, protein, fat, and fiber content resulted in improved predictive ability compared with previous predictive equation estimates for ME content of pet foods.

The variance in the modified Atwater and NRC predictive equations when compared to actual measured ME concentrations results from the error associated with energy digestibility. For example, the reduced energy digestibility found in canned foods results in an overestimate of ME concentration when using the NRC and modified Atwater factors predictive equations, whereas the more digestible dry foods had a slight underestimation of ME concentration using these predictive equations. Thus, dry foods were slightly more digestible than canned foods or foods used to derive the predictive equations. There was a significant benefit to using measured GE as a term in the new predictive equations (as compared to the NRC equation which predicts GE) and generating new coefficients associated with moisture, protein, fat and fiber content. These changes improved the predictive equation such that there was no difference between the average of the estimated ME concentration and the average of the measured ME concentration. Precision was also improved by using the new equation for ME content, evidenced by the higher r^2^ and smaller numbers for the absolute value of the difference between measured and estimated ME concentrations. Because the mean value of the absolute difference between measured and estimated ME concentrations for all foods was less than 2.5% of the measured ME when using the new equations that include a term for measured GE, it is likely that for most foods a calculated ME is within measurement error of the actual ME concentration.

The second objective of this retrospective study was to examine the effects of nutrient digestibility on stool quality. In both dogs and cats, we found that stool quality was better (higher fecal grade score) when they were fed foods that had lesser amounts of protein or greater amounts of fiber passing into the large intestine. These data support the hypothesis that microbial changes associated with increased protein in the large bowel include increased numbers of proteolytic bacteria, which result in increased production of ammonia and sulfur containing compounds that are detrimental to bowel health. Microbial population shifts in response to dietary protein load have been demonstrated in cats [16] and dogs [17], [20]. There was also a positive benefit for dogs and cats on stool quality if foods had greater amount of fiber delivered to the large bowel. The improvement in stool quality associated with more fiber in the large intestine may again be attributed to a change in microbial populations within the large intestine, or it may be the result of a change in transit time through the large intestine. Dietary fiber has been shown to alter large bowel transit time in dogs [21]. The effects of dietary fiber on stool quality may be related to the length of cellulose fiber rather than the absolute amount of fiber [18]. Similarly, in cats the addition of dietary fiber in the form of long-fiber cellulose enhances stool quality [19]. Thus, not only the amount of fiber delivered to the large bowel, but also the type of fiber is important for stool quality.

The clinical utility of these findings are two fold. First, these new ME predictive equations for dog and cat pet foods that utilize measured GE provide more accurate estimates of ME concentration in foods. Thus, foods can be offered in amounts that are less likely to result in overfeeding, obesity, and its associated disorders. Second, in dogs or cats with poor stool quality, feeding foods with lesser amounts of undigestible protein or greater amounts of fiber passing into the large intestine will augment other therapies aimed at treating the underlying cause of poor stool quality.
